# Glaucoma in Ectropion Uveae Syndrome: A Case Report and Literature Review

**DOI:** 10.18502/jovr.v14i3.4793

**Published:** 2019-07-18

**Authors:** Mohammadmehdi Hatami, Azadeh Doozandeh, Mohadeseh Feizi

**Affiliations:** Ophthalmic Research Center, Shahid Beheshti University of Medical Sciences, Tehran, Iran

## Abstract

**Purpose:**

To report a case of advanced childhood glaucoma secondary to congenital ectropion uveae (CEU).

**Case Report:**

The patient was a seven-year-old boy with unilateral glaucoma secondary to CEU and facial asymmetry, mild unilateral ptosis, and proptosis in the left eye. The intraocular pressure (IOP) was 28 mmHg and cup-to-disc ratio was 0.8 in the left eye. After starting glaucoma medication, IOP decreased to 21 mmHg. In view of the uncontrolled IOP with medication and high cup-to-disc ratio and increased axial length of the left eye, mitomycin-C (MMC)-augmented trabeculectomy was planned. Despite sub-tenon MMC injection and bleb needling, the bleb failed after six months, and we had to perform a shunt procedure to control the IOP.

**Conclusion:**

Although CEU is rare, ophthalmologists should be familiar with this syndrome because of the high frequency of glaucoma and its challenging management during childhood.

##  INTRODUCTION

Ectropion uveae (EU) is defined as the presence of iris pigment epithelium on the anterior surface of the iris. Although EU is usually acquired, it may occur as an isolated congenital anomaly or in association with systemic diseases such as neurofibromatosis.

The most important complication of EU is glaucoma, and its diagnosis and treatment are challenging during childhood because the intraocular pressure (IOP) cannot be easily measured in a crying toddler under routine settings, and significant damage may have already occurred by the time of presentation. Hence, early detection, close monitoring, and prompt treatment are mandatory to safeguard lifelong vision of the child.

We report a case of glaucoma secondary to EU and its management, along with a literature review.

##  CASE REPORT

A seven-year-old boy was referred to glaucoma service for mild ptosis and gradual decrease in the left eye vision.

**Figure 1:**
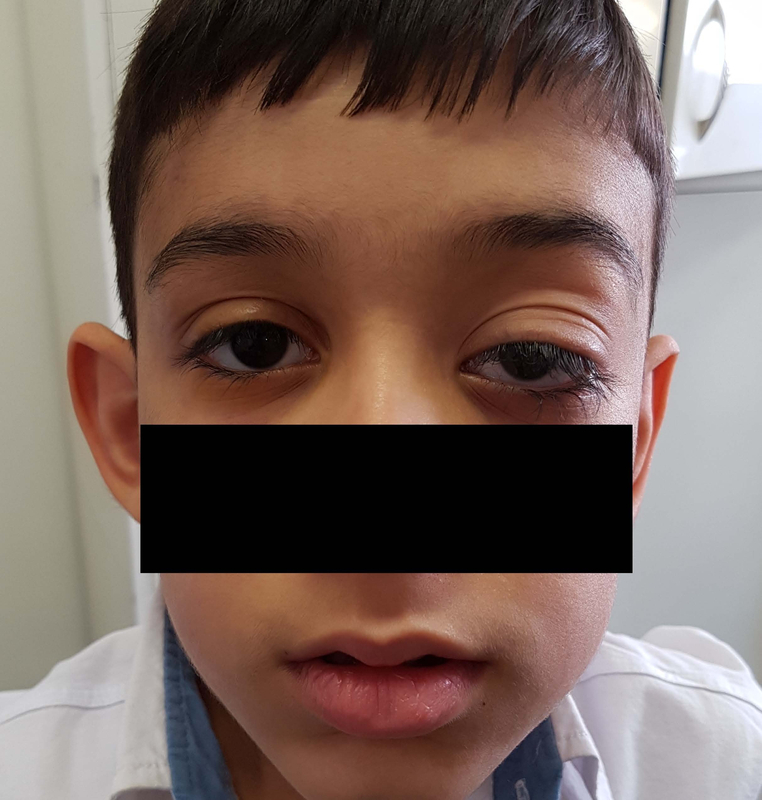
General appearance of the patient. Mild ptosis and facial hemihypertrophy is seen.

He had facial asymmetry, with left hemifacial hypertrophy and moderate ptosis and proptosis of the left eye [Figure 1]. There was no history of any medical or surgical intervention; family history was unremarkable. Best-corrected visual acuity were 20/20 and 20/30 in right and left eyes, respectively. Full cycloplegic refraction was +1.75 diopters in the right eye and -3.5 diopters in the left eye. Relative afferent pupillary defect was +1 in the left eye. There was 2 mm ptosis in the left eye with normal levator palpebrae function. Hertel exophthalmometry measurements were 14 and 21 mm with bar reading of 107 mm. The axial length was 22.21 and 24.51 mm in the right and left eyes, respectively. Extraocular muscle movement was normal.

**Figure 2:**
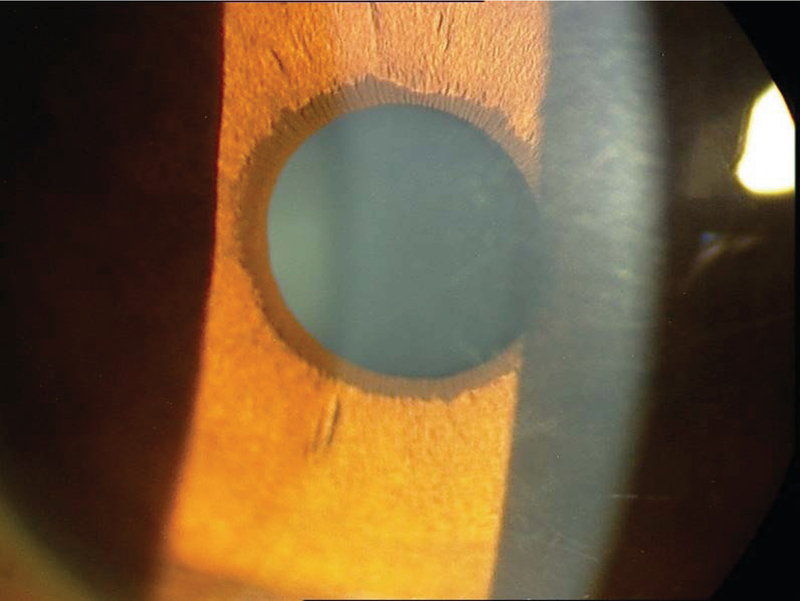
Slit lamp view shows ectropion uvea in left eye. Right eye is normal.

On slit lamp examination, the cornea was clear and the anterior chamber (AC) depth was normal. The iris was cryptless and there was a band of hyperpigmentation at the margin of the pupil consistent with EU [Figure 2]. Gonioscopy showed anterior iris insertion and angle dysgenesis. Since EU may be associated with systemic disorders, the whole body was examined carefully to detect any remarkable signs of probable associated diseases. Skin examination was normal, and there was no café-au-lait spot or any other sign of neurofibromatosis. Dental examination was also normal. His height and weight measurements were within normal limits for his age. His mental and intellectual abilities were age-appropriate.

**Figure 3:**
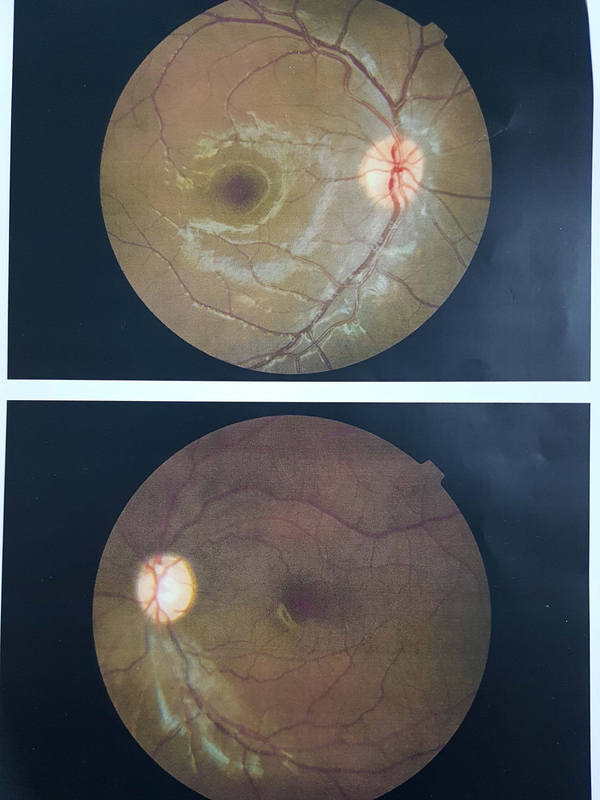
Fundus photograph shows advanced cupping of left optic disc.

**Figure 4:**
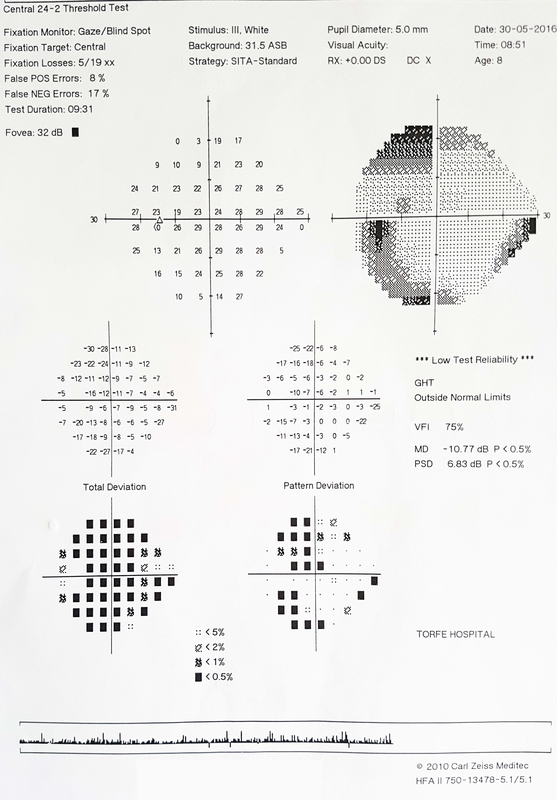
Perimetry shows double arcuate scotoma in left eye.

**Figure 5:**
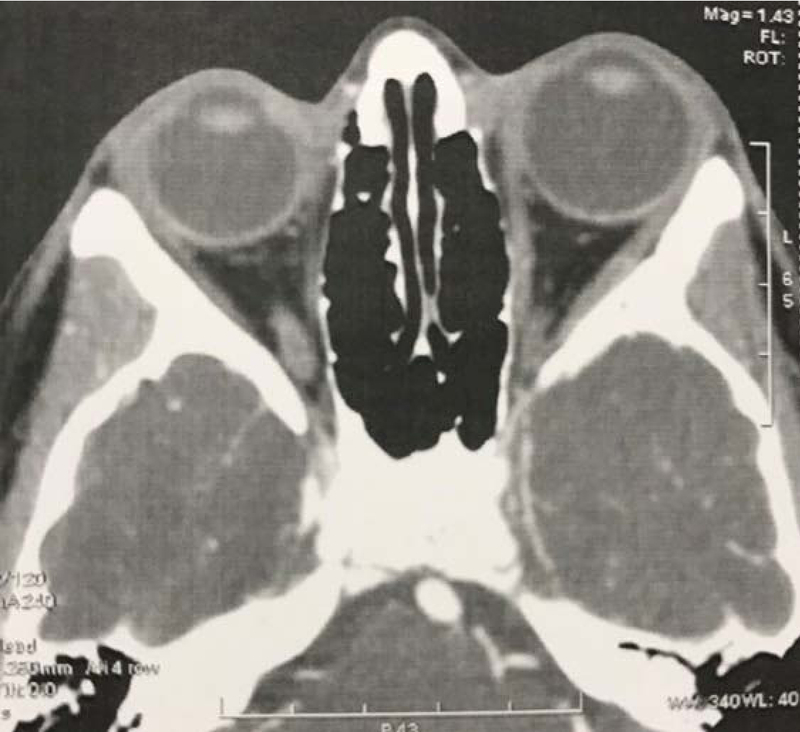
Axial computerized tomography (CT) scan shows left proptosis and different globe size.

IOP was 13 and 28 mmHg without medication in the right and left eyes, respectively. After starting three glaucoma medications for the left eye, IOP decreased to 21 mmHg. Cup-to-disc ratio was 0.2 and 0.8 in the right and left eyes, respectively, with significant rim loss in the left eye [Figure 3]. Horizontal corneal diameter was 12 and 12.5 mm, and central corneal thickness was 578 and 589 μm in the right and left eye, respectively. Peripapillary nerve fiber layer optical coherence tomography (OCT) and perimetry were normal in the right eye, but in the left eye there was severe nerve fiber layer loss in all quadrants in peripapillary OCT and a double arcuate scotoma in perimetry [Figure 4]. Axial and coronal orbital computerized tomography (CT) scan showed left proptosis and axial elongation of the left globe [Figure 5].

**Figure 6:**
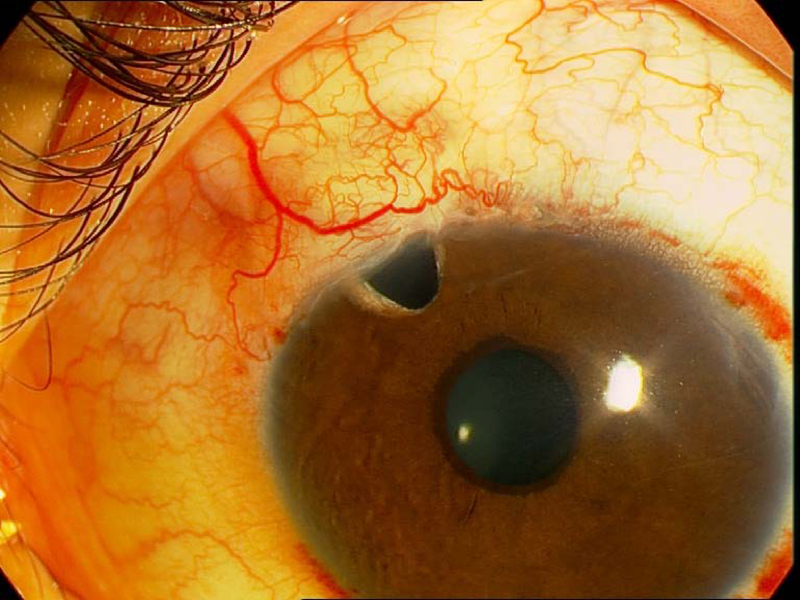
Slit lamp photograph one month after trabeculectomy. The bleb is shallow and moderately vascularized.

Due to asymmetric corneal diameter and antimetropia, the clinical impression was that the glaucoma in this patient was of early onset and has been missed; hence, prompt intervention was crucial. The patient was planned for Mitomycin-C (MMC)-augmented trabeculectomy, which was performed without complications. At the one-month postoperative examination, IOP was 17 mmHg without medication, and the bleb was moderately vascularized and shallow [Figure 6]. Although subtenon MMC was injected in the superior fornix, the bleb failed after six months, and IOP increased to 20 mmHg. Bleb needling and MMC injection were not successful, and we had to perform a shunt procedure to control the IOP. Six months after the shunt procedure, IOP was controlled with the timolol-dorzolamide fixed combination.

##  DISCUSSION

Ectropion uveae is characterized by the presence of iris pigmented epithelium on the anterior surface of the iris. It can be acquired, associated with systemic diseases, or can manifest itself as an isolated congenital syndrome. Acquired EU may be associated with any inflammatory, ischemic, or neoplastic process involving the iris such as neovascular glaucoma or iridocorneal endothelial (ICE) syndrome. This type of ectropion is commonly progressive. EU can be associated with systemic diseases, such as: neurofibromatosis type 1 (NF1), Prader-Willi syndrome, and Rieger syndrome.

Ritch et al reported eight patients with CEU and glaucoma, of which three had NF1.^[1]^ Morales et al found glaucoma in 13 of 56 patients with NF1 with orbital involvement, and eight of these thirteen cases had EU.^[2]^ In another study, all five patients with orbitofacial NF1 and glaucoma demonstrated histopathological EU.^[3]^ So, the presence of EU in a neonate necessitates a workup for NF.^[4]^ Isolated CEU syndrome is usually unilateral and tends to be non-progressive. It is characterized by iris pigment hyperplasia onto the anterior surface of the iris around the pupillary margin. In contrast to acquired EU, the iris sphincter muscle and stroma are not affected in CEU and are not everted on histopathologic examination.^[5]^

The iris-pigmented epithelium hyperplasia is thought to be induced by an embryological remnant that fails to fully regress in the AC.^[6]^ This anomaly may be caused by a late developmental arrest of neural crest tissue in utero.^[5]^

Typical clinical findings include a smooth and cryptless iris surface, proliferation of iris pigment epithelium onto the anterior surface of the iris, and glaucoma. Gonioscopy typically reveals anterior iris insertion, angle dysgenesis, and incomplete formation of trabecular meshwork and Schlemm's canal,^[1,5,6]^ which is the primary mechanism of glaucoma in CEU.

Glaucoma is a frequent complication of CEU. In one report, glaucoma occurred in seven of eight cases,^[1]^ and in another study, in nine of ten CEU cases.^[5]^ So, all patients with CEU should be evaluated periodically to detect and treat glaucoma.^[1,5]^ The affected eye may exhibit mild ptosis with good levator function. This finding is most likely related to the neural crest origin of Mueller's muscle. In the aforementioned study, ptosis was reported in one of eight cases^[1]^ and in six of ten cases with CEU.^[5]^

Our patient also had antimetropia in cycloplegic refraction because of greater axial length in the left eye. There was combined pseudo and true proptosis, because the amount of proptosis of the left eye on Hertel exophthalmometry was greater than the axial length difference, and hemifacial hypertrophy seemed to play a role in proptosis. This finding also presented in case 1 of a study by Bansal et al,^[7]^ who reported 6 mm proptosis and 2.2 mm difference in axial length. Although they did not mention combined mechanism proptosis, the report was similar to the present study.

In the approach to a patient with CEU and glaucoma, we should consider diseases that commonly mimic it, such as Axenfeld-Rieger syndrome (ARS), iridocorneal endothelial (ICE) syndrome, and NF1. In ARS, the findings include a high iris insertion, angle dysgenesis, EU, and glaucoma. However, the syndrome can be distinguished from CEU using other clinical findings such as posterior embryotoxon, corectopia, or polycoria, and facial abnormalities such as maxillary hypoplasia and dental defects.^[8]^ ICE syndrome is an acquired condition with glaucoma and EU. ICE syndrome predominantly affects middle-aged women, and its occurrence in children is rare.^[9]^

The treatment of glaucoma in patients with CEU usually requires surgery, although medical management should be attempted initially. Effective surgical interventions for CEU-associated glaucoma differ from the techniques used in primary congenital glaucoma, and the success rate is much lower, presumably due to severe angle dysgenesis in this disorder. Glaucoma filtration surgery or a shunt procedure may be necessary.^[8,10]^ In one study, a total of six goniotomies in three patients with glaucoma associated with CEU did not control IOP, and they finally required trabeculectomy.^[5]^ Early failure of trabeculectomy in our patient might suggest prompt consideration of a shunt procedure in CEU-associated patients, when IOP is not controlled with medical treatment.

In conclusion, although CEU is a rare syndrome, it is associated with systemic diseases and high frequency of glaucoma and its management in children is challenging. Hence, ophthalmologists should be familiar with this rare syndrome to prevent irreversible vision loss.

###  Declaration of Patient Consent

The authors certify that they have obtained all appropriate patient consent form. In the form, the patient has given his consent for his images and other clinical information to be reported in the journal. The patient understands that his name and initials will not be published and due efforts will be made to conceal his identity, but anonymity cannot be guaranteed.

##  Financial Support and Sponsorship

Nil.

##  Conflicts of Interest

There are no conflicts of interest.
